# Daylight saving time, circadian rhythms, and cardiovascular health

**DOI:** 10.1007/s11739-018-1900-4

**Published:** 2018-07-03

**Authors:** Roberto Manfredini, Fabio Fabbian, Rosaria Cappadona, Pietro Amedeo Modesti

**Affiliations:** 1 Department of Medical Sciences, University of Ferrara, Via Fossato di Mortara 64/B, 44121 Ferrara, Italy; 2 Department of Morphology, Surgery and Experimental Medicine, University of Ferrara, Via Luigi Borsari 46, 44121 Ferrara, Italy; 3 Department of Clinical and Experimental Medicine, University of Florence, Largo Brambilla 3, 50134 Florence, Italy

**Keywords:** Daylight saving time, Circadian rhythms, Chronobiology, Myocardial infarction, Gender, Sleep deprivation, Chronotype, Climate

## Abstract

Very recently, the European Parliament, called to decide on possible abolition of the Daylight Saving Time (DST), approved a resolution calling the scientific community to conduct a more in-depth evaluation. The question is based on disruption of body’s circadian rhythms. We review here the relationship between DST and cardiovascular health. The available evidence suggests the existence of an association between DST and a modest increase of occurrence of acute myocardial infarction, especially in the first week after the spring shift. Possible mechanisms include sleep deprivation, circadian misalignment and environmental conditions. The role of gender and individual preference in circadian rhythms (chronotype) will need further assessment.

## Background

For many years, clocks in the countries of the European Union (EU) have been put forward by 1 h on the last weekend of March, to achieve the daylight saving time (DST). Several months ago, Finland called for the abolition of DST across the EU based on concerns for human health. The European parliament approved a resolution (384 votes in favour, 153 against, 12 abstentions) calling for the European commission and the scientific community to conduct a more-in-depth “full evaluation.”

Changing the time disrupts our body clocks. For most people, changing the time—even if only by 1 h—may produce tiredness as a small inconvenience. Other people, however, can have more serious consequences. The complex interaction among circadian body clocks and health represents a broad topic, explored by a growing body of literature, which cannot be reviewed in this study. We aim to provide information on circadian rhythms, their regulation and disruption, and possible effects on the cardiovascular (CV) system.

## Circadian rhythms

All living organisms are characterized by multiple mechanisms to favour the synchronization and adaptation to the environment and its variations, and rhythmic organization represents a pivotal mechanism. Biological rhythms exist at any level of living organisms and, according to their cycle length, may be classified by Latin terms into the following: (a) circadian rhythms (from ‘circa-diem’: period of approximately 24 h), (b) ultradian rhythms (‘ultra-diem’: period shorter than 24 h, e.g., hours, minutes, and seconds), and (c) infradian rhythms (‘infra-diem’: period longer than 24 h, e.g., days, weeks, and months) [1].

Circadian rhythms, the most widely studied, are driven by either central or peripheral clocks. The central circadian clock (called ‘master clock’) is located within the suprachiasmatic nucleus (SCN) of the hypothalamus and accounts for approximately 20,000 neurons. Circadian clocks roughly consist of a set of proteins, capable of generating self-sustained positive and negative transcriptional feedback loops with a free-running period of approximately 24 h. However, peripheral circadian clocks have also been found within the great majority of mammalian cells and tissues. The central clock and the peripheral clocks can directly and indirectly influence numerous functions, including sleep/rest and locomotor activities, feeding and drinking behaviour, core body temperature, endocrine activity, metabolism, autonomic and sympathetic activity, and many others [2]. The CV system has biological clocks, as well. In fact, biological clocks have been found in the heart (at the cardiomyocyte level), blood vessels, and vascular endothelial cells. For example, the circadian clock within cardiomyocytes directly regulates myocardial metabolic gene expression, most likely in anticipation of the sleep/wake and feeding/fasting cycle. Thus, the results obtained on murine models show that the circadian clock within the cardiomyocytes allows for the rapid adaptation of the heart to the prolongation of fasting. Thus, a desynchronization of an organism with its environment, through circadian clock derangements, may also lead to the development of CV diseases [2].

## Disruption of circadian rhythms

Circadian clocks regulate metabolism in many systems and organs, including the heart, skeletal muscle, liver, and adipose tissue. In particular, the cardiomyocyte circadian clock is essential for the responsiveness of the heart to fatty acids. When the cell cannot face increased fatty acid availability due to its inability to adequately increase fatty acid utilization, an accumulation of detrimental intracellular long-chain fatty acid derivatives occurs. This accumulation, occurring via multiple mechanisms, may affect the contractile function of the heart, as well as a series of other components, such as glucose intolerance, insulin resistance and insufficiency, dyslipidaemia, and increased vascular resistance [2]. Conversely, the circadian clock within the heart is altered in various animal models of human disease, including hypertension, diabetes, myocardial infarction, and simulated shift work.

Shift work, a circadian misalignment resulting from a 12-h inversion of the behavioural cycle (including sleep/wake and fasting/feeding cycles), is a typical example of rhythm disruption. Shift workers are exposed to a misalignment of their behavioural and environmental cycles, which is a risk factor for hypertension, inflammation, and CV disease. It has been observed that compared with circadian alignment, circadian misalignment can (a) increase both systolic and diastolic blood pressure (BP); (b) decrease heart rate (HR) during wake periods and increase HR during sleep time; (c) reduce the sleep opportunity-associated dip in BP and HR; (d) affect the 24-h urinary epinephrine and norepinephrine excretion rates; (e) decrease markers of cardiac vagal modulation; (f) increase inflammatory markers, particularly 24-h interleukin-6 (IL-6), 24-h C-reactive protein (CRP), resistin, and tumour necrosis factor (TNF); and (g) decrease plasminogen activator inhibitor-1 (PAI-1) levels. Taken together, all these effects can explain the increased prevalence of CV disease in night workers versus day workers [3].

## Daylight saving time: an insidious form of disruption?

**Fig. 1:**
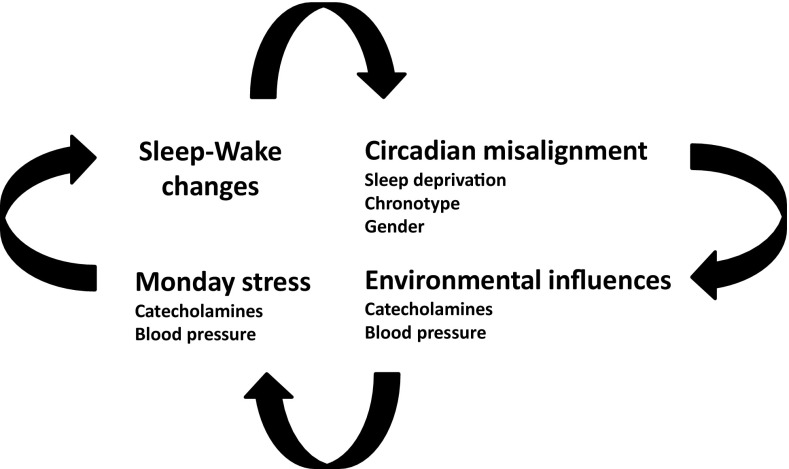
Schematic representation of factors potentially contributing to a higher cardiovascular risk following the daylight saving time (DST) shift

DST refers to the practice of adjusting the local clock time. In 1784, Benjamin Franklin first analysed the cost of candle consumption during dark evenings in Paris, but, only in 1916, DST was adopted in Great Britain due to the costs of energy during the war. Despite common opinion that the DST spring shift leads to the relatively inconsequential loss of 1 h of sleep on the same night, increased sleep fragmentation and sleep latency present a cumulative effect of sleep loss at least across the following week, possibly longer (Fig. 1). However, the autumn transition is simply considered to be a gain of 1 h of sleep. Again, there is a little evidence of extra sleep on that night, and the cumulative effect of 5 consecutive days of earlier rise times following the autumn change suggests a net loss of sleep across the week. In any case, short sleepers are more disadvantaged in their efforts to adjust to the clock change in both spring and autumn [4].

Circadian clocks use daylight to entrain to the organism’s environment. Entrainment is sufficiently exact that humans adjust to the east–west progression of dawn within a given time zone. Similar to other animals, humans are seasonal, although seasonality in humans has drastically declined in industrialized countries over the last 60 years. DST might constitute an additional factor for the dissociation of human biology from the seasons [5]. However, since studies on the effects of DST transitions essentially investigate the potential re-entrainment of individuals to a new social schedule, it is important to consider the individual’s circadian preference (chronotype), which can exhibit substantial variations within a given population. In fact, depending on genotype, gender, age, and light exposure, individual clocks will adopt a different phase relationship to dawn.

## A preferred high-risk time for unfavourable cardiovascular events?

The CV system is organized according to a specific oscillatory order, and most CV functions exhibit circadian changes. Thus, circadian differences in the physiological status of the CV system give rise to rhythmic—and predictable-in-time—variations either in the susceptibility of human beings to morbid and mortal events, or in the ability to precipitate the overt expression of disease [1]. It is known that the occurrence of CV events exhibits peculiar temporal patterns that vary across 24 h of the day. These patterns coincide with the temporal variation in the (a) pathophysiological mechanisms that trigger CV events and (b) physiological changes in the body rhythms. A series of factors, not harmful if considered alone, are capable of triggering unfavourable events when they all occur within the same temporal window [1]. An evident circadian (morning) rhythm for acute myocardial infarction (AMI) has been understood for more than 2 decades. It has been estimated that the incidence rate of AMI onset is 40% higher in the morning period than throughout the rest of the day, and nearly 28% of morning AMIs (accounting for approximately 9% of all AMIs) are attributable to the morning excess [6]. Moreover, rupture or dissection of aortic aneurysms shows a circadian variation, with an evident morning preference, as well [7]. The similarity of temporal patterns of such different acute CV and cerebrovascular events suggests that they share common underlying mechanisms. Several pathophysiologic phenomena, such as increased BP, HR, sympathetic activity, basal vascular tone, vasoconstrictive hormones, increased viscosity, fibrinogen, platelet aggregability, and reduced fibrinolytic activity, exhibit prominent circadian rhythms with phases positively correlated with the timing of excess in CV events [7]. Moreover, the effect of stress on human cardiac, vascular, and haemostatic status appears to be circadian rhythmic, with the effects being greater in the morning [1]. An exaggerated CV response to behavioural challenges has been suggested as a potential factor that enhances the risk of hypertension and CV disease. Thus, individuals who exhibit large increases in BP during acute psychological stress are at risk of atherosclerosis, probably secondary to endothelial damage by a BP surge, via increased flow turbulence, and a subsequent release of inflammatory and pro-atherogenic cytokines. Emotional stress can also play a pivotal triggering role for ischaemic heart disease, as shown for the morning onset of Takotsubo syndrome [8].

## Daylight saving time and acute myocardial infarction: does an association exist?

Janszky and Ljung first reported a higher incidence of AMI following the spring DST shift, which was more pronounced in women [9]. We reviewed the available literature on this association [10], and found another six studies, four conducted in Europe and two in the United States, accounting for a total of 87,994 cases. Although there were differences among each other, they all supported the existence of an association between DST and the risk of AMI, particularly after the spring DST transition, with an increase ranging from 4 to 29%. In particular, three studies report a higher incidence on Monday, and only four provide an analysis by separating subgroups by gender [10].

### Sleep deprivation

The central master clock is primarily entrained by light, and reduced exposure to light during the day and over exposure to light at night due to artificial lighting may impair the circadian organization of sleep. Changes in sleep architecture during sleep disruption may lead to increased energy intake, reduced energy expenditure, and insulin resistance. Short (< 6 h) sleep has been associated with negative health outcomes including hypertension, diabetes mellitus, obesity, and even mortality. A recent study has shown that short sleep, compared with normal sleep, is associated with a significant increase in the relative risk (RR) of mortality due to all causes (RR 1.12). Moreover, short sleepers have increased rates of obesity (38%), diabetes mellitus (37%), coronary heart disease (26%), and hypertension (17%) [11].

### Personal circadian preference (chronotype) and gender

In 1976, Horne and Ostberg published a milestone study on possible individual differences in circadian attitudes in the International Journal of Chronobiology. By means of a simple self-assessment questionnaire (Morningness–Eveningness Questionnaire, MEQ), they evaluated individual differences in the circadian variation of oral temperature. Subjects categorized as morning types (M-type) show a significantly earlier peak time than evening ones (E-type) and have a higher daytime temperature. Intermediate types (I-type) have temperatures between those of the other groups. Recently, we collected the available evidence dealing with the association between chronotype, gender, and general health [12]. Individuals with the evening chronotype show sleep-related issues, e.g., later bedtime and wake-up and decreased sleep quality and quantity. Moreover, these people are prone to skipping breakfast, to have reduced physical activity, unhealthy dietary patterns, and other habits (smoking and alcohol), along with poor glycaemic control, metabolic syndrome, and diabetes. There are conflicting findings on whether men and women differ in their energy balance responses to sleep disruption, but women seem to be more susceptible. In particular, the female–evening association gives rise to several problems. Excessive daytime sleepiness and poor sleep quality are higher among females, and female predominance in the rate of depression is observed in subjects with a delayed sleep–wake schedule. Moreover, disturbances of the sleep–wake cycle in E-type females can also be increased by the presence of nightmares. Evening association may also be linked to being overweight, a feature that may more significantly impact the emotional health of adolescent females. Chronotype may also influence cognitive patterns and academic achievement. Evening association, in fact, can be a problem for morning attention at school, which is more evident in E-type girls compared to E-type boys [12].

## Does the climate have a role in daylight saving time-linked CV risk?

The external influences of the environment (mainly climate) on biological rhythms are commonly observed by physicians. In clinical practice, it is common to find patients with mild hypertension requiring pharmacological treatment only in the winter, while, in the summer, they can suspend or reduce drug intake. Public health providers are aware of the 5–30% excess of mortality experienced in winter by most countries, mainly due to cerebrovascular events, and frail elderly often perceive the end of winter as a triumphant passage of Cape Horn [13]. Likewise, following the pioneering observation of the Medical Research Council’s trial [14], where seasonality is associated with larger BP variations than those induced by drugs, researchers have now found that a clinical trial should last at least 1 year to mitigate the bias introduced by seasonality. Finally, although epidemiologists are aware that the hypertension burden assessed in large surveys may provide different results by season, the importance of environmental variations on health sometimes remains neglected.

It is a common opinion that the main consequences of the spring DST are mediated by changes in sleeping time. To modulate this effect, the change in the local clock time is usually made on the night between Saturday and Sunday. Interestingly, as considered above, several studies report the highest incidence on Monday or at least in the first working days of the week [10]. One day (Sunday) is clearly insufficient to become accustomed to waking up an hour earlier in the morning. However, the phenomenon can be seen from a different point of view, and other actors could play a role in increasing the risk of AMI or stroke. During spring, a complex interaction exists between single variables such as environmental parameters (i.e., temperature or light exposure), behavioural factors (such as physical activity amplitude, type, and intensity), and DST initiation, and these single variables may mutually influence each other. Why should this complex interaction increase CV risk? Although lowering of temperature is commonly considered responsible for an increase in BP, the first risk factor for stroke, data from a population-based study including 45,787 ischaemic stroke patients in Tuscany (Italy) from 1997 to 2007, shows that every degree celsius increase in temperature difference over 24 h is associated with a significant 1.5% increase in ischaemic stroke admissions [15]. A simplistic reading of this relationship could suggest a direct role of increase, rather than decrease, temperature in stroke. However, the association is more complex. The marked increase of stroke hospitalizations observed in Tuscany when the ΔT850hPa exceeds 5 °C apparently is not observed with the significant increase of all strokes observed in the same study when the T850hPa decreases [15]. This apparent contradiction is solved knowing that most of the ΔT850hPa increases exceeding 5 °C (94% of days) occur during the coldest months of the year (October–March) and never during warmer months (June–September). This finding means that stroke increases when a substantial increase of ΔT850hPa starts from a relatively low daily average temperature value, typical of the coldest months of the year. Furthermore, all studies refer to air temperature measured outdoors, while subjects live in heated houses. These day-to-day temperature changes may thus be associated with behavioural changes that might, in turn, influence the risk of stroke hospitalizations. In spring, when the temperature rises, people spend more time outdoors, and those at risk can suffer the greatest consequences.

## Conclusions

The results of this study suggest an association between DST and a modest increase of AMI occurrence, even if only after the spring shift. Furthermore, in contrast to earlier findings, a higher risk is not confirmed for women. On one hand, it is possible that, after the spring DST shift, even modest sleep deprivation and circadian misalignment may affect CV health, since it has been associated with increases in sympathetic tone and catecholamine levels. On the other hand, it is known that most people adjust more readily to delays than to advances, i.e., they suffer less from jet lag after westward than after eastward flights. A similar pattern has been suggested for DST transitions. Moreover, adjustment to DST transitions is chronotype specific, and differences are more marked after the spring shift [5]. Thus, further studies should consider the assessment of individual chronotype, sleep characteristics, and gender. Unfortunately, less than 44% of recent studies on the time of onset of CV events provide separate analysis [16]. Finally, in some studies, Monday exhibits the highest frequency of AMI onset. This finding is not surprising, since Monday is a critical day for the onset of AMI, stroke, and Takotsubo cardiomyopathy [17–19], probably due to a series of unfavourable factors, including the stress of commencing weekly activities and higher catecholamine and BP levels.

Furthermore, mechanisms other than rhythm disruption may also play a role. In spring, when the temperature rises, people spend more time outdoors, and those at risk may suffer the greatest consequences. Therefore, it cannot be excluded that the excess CV risk observed on Monday after the DST spring shift may be attributable to the anticipated exposure to the outdoor environment. In this condition, the thermal perception of people might be especially worsened if subjects are surprised in the outdoor environment with inappropriate clothing. Correct clothing behaviour was, indeed, reported to contribute to preventing excess winter mortality [20]. The importance of clothing may be particularly relevant to low-income families and other economically vulnerable persons who are disproportionately exposed to cold temperatures. Waiting for further in-depth studies and decisions by the European lawmakers, and prior to drastically moving towards DST abolition, a smoother approach could be suggested. As we now, discourage males over 55 years old with hypertension, obesity, and metabolic syndrome to perform their daily jogging early in the morning, providing adequate information to the population, especially to the highest-risk individuals, could help. The following is an easy strategy: (a) move bedtime 1 h a few days prior to the spring shift, to limit sleep deprivation effects; and (b) take care in exposing oneself to abrupt changes of temperature in the immediate post-shift days. Could such an easy combination of sleep strategies, scarves, hats, and gloves effectively reduce the CV effects of DST?
